# Supplementary material for: Free mobility across group boundaries promotes intergroup cooperation
Source: Commun Psychol. 2025 Jan 25;3:10. doi: 10.1038/s44271-025-00192-y (PMC11762412; doi:10.1038/s44271-025-00192-y)
Supplement: Supplementary file 2 — Supplementary Information [file 44271_2025_192_MOESM2_ESM.pdf]

Supplementary Information

Free mobility across group boundaries promotes intergroup cooperation

Jörg Gross, Martin Götz, Katharina Reher, Filippo Toscano

Supplementary Note 1–7
Figures S1–S7
Tables S1–S18

Supplementary Note 1. Analyses

All statistical analyses were performed using R (version 4.2.2). Since participants made repeated decisions in fixed groups, we fitted multilevel regression models (as implemented in the lme4 package, version 1.1-35.5) using two nested random intercepts (i.e., modelling repeated individual decisions nested in individuals, and groups). For cooperation (i.e., stage 1) and 'helping' choices (i.e., stage 2), we fitted logistic multilevel regressions, since the dependent variable was binary. Thus, estimates (*b*) represent log-odds. For the post-task self-reports on identification, we aggregated the data to the subject-level (since we only have one observation per subject, in this case), and fitted a linear multilevel regression model with one random intercept, estimating the respective group-level average.

Supplementary Note 2. Choices across rounds

In addition to what is reported in the main manuscript, figures S1–S3 illustrate choices to keep the token, round earnings, and overall helping frequency across rounds and per treatment, respectively. Tables S1–S3 show the results of cooperation/keeping choices across treatments.

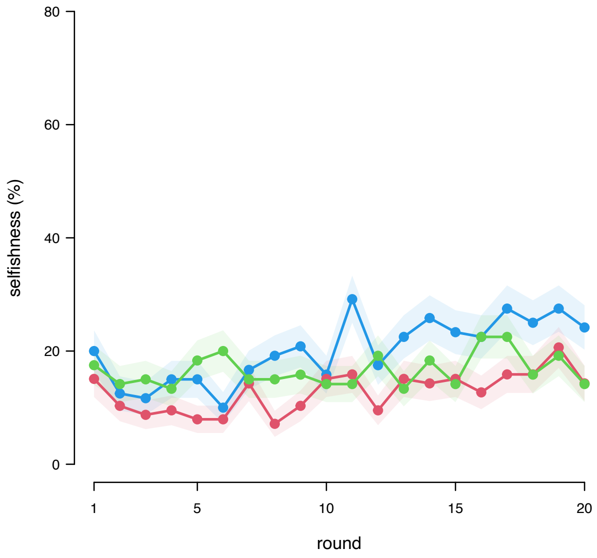

Figure S1. Selfishness. Average proportion of keeping the resource at stage 1 (i.e., not cooperating) across rounds in the restricted- (red), forced- (blue) and free- (green) mobility treatment. Colored bands indicate the standard errors of the round means based on n = 120 observations per treatment and round.

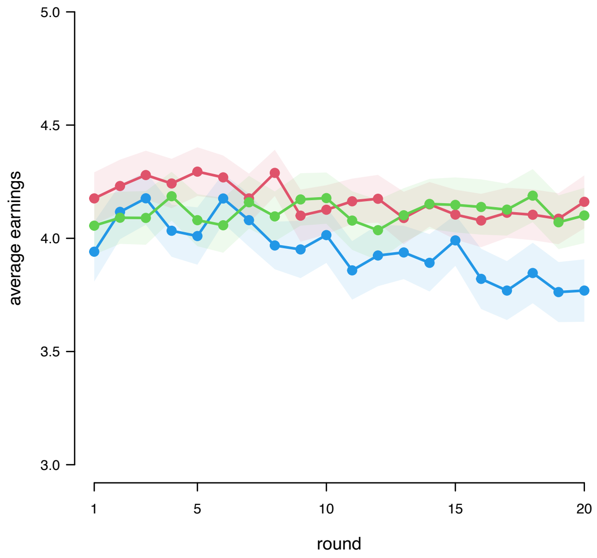

Figure S2. Earnings. Average round earnings in the restricted- (red), forced- (blue) and free- (green) mobility treatment. Colored bands indicate the standard errors of the round means based on n = 120 observations per treatment and round.

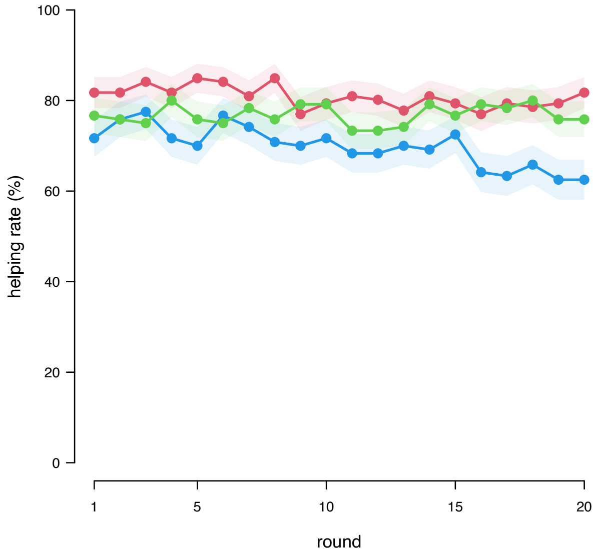

Figure S3. Helping. Average helping rate across rounds in the restricted- (red), forced- (blue) and free- (green) mobility treatment. Colored bands indicate the standard errors of the round means based on n = 120 observations per treatment and round.

Table S1. Differences in group cooperation (stage 1) across treatments and rounds.

	b	CI	SE	z	p	
Intercept (forced)	-1.223	[-1.734;-0.712]	0.261	-4.692	<.001	***
Restricted	0.788	[0.077;1.498]	0.362	2.173	.030	*
Free	0.269	[-0.450;0.988]	0.367	0.734	.463	
Round	-0.025	[-0.043;-0.007]	0.009	-2.701	.007	**
Restricted × round	0.050	[0.025;0.075]	0.013	3.981	<.001	***
Free × round	-0.013	[-0.038;0.012]	0.013	-1.005	.315	
**Post hoc comparisons**						
Free × round - restricted × round	-0.063	[-0.087;-0.039]	0.012	-5.058	<.001	***

Note. Logistic multilevel regression; dependent variable: 1 = group cooperation; * <.05, ** <.01, *** <.001

Table S2. Differences in intergroup cooperation (stage 1) across treatments and rounds.

	b	CI	SE	z	p	
Intercept (restricted)	-0.112	[-0.764;0.539]	0.332	-0.338	.735	
Forced	0.561	[-0.371;1.494]	0.476	1.180	.238	
Free	0.129	[-0.802;1.059]	0.475	0.271	.786	
Round	-0.058	[-0.076;-0.04]	0.009	-6.220	<.001	***
Forced × round	0.030	[0.005;0.056]	0.013	2.315	.021	*
Free × round	0.088	[0.062;0.113]	0.013	6.731	<.001	***
**Post hoc comparisons**						
Round + free × round	0.03	[0.012;0.047]	0.009	3.263	.001	**

Note. Logistic multilevel regression; dependent variable: 1 = universal cooperation; * <.05, ** <.01, *** <.001

Table S3. Differences in keeping (stage 1) across treatments and rounds.

	b	CI	SE	z	p	
Intercept (restricted)	-4.734	[-5.62;-3.847]	0.452	-10.463	<.001	***
Forced	1.197	[0.037;2.358]	0.592	2.022	.043	*
Free	1.267	[0.098;2.436]	0.596	2.124	.034	*
Round	0.065	[0.037;0.094]	0.015	4.459	<.001	***
Forced × round	0.012	[-0.025;0.049]	0.019	0.656	.512	
Free × round	-0.048	[-0.086;-0.009]	0.020	-2.428	.015	*

Note. Logistic multilevel regression; dependent variable: keep; * <.05, ** <.01, *** <.001

Supplementary Note 3. Choice to meet out-groups

Table S4 shows the regression results on the reported model explaining stage 2 decisions to meet out-groups in the free-mobility treatment depending on choices in stage 1.

Table S4. Choice to meet out-group members (stage 2) depending on own cooperation decision and feedback on collective choices in stage 1.

	b	CI	SE	z	p	
Intercept (own stage 1 choice: group coop.)	-1.073	[-1.642;-0.504]	0.290	-3.697	<.001	***
Own stage 1 choice: keep	0.465	[-0.292;1.221]	0.386	1.204	.229	
Own stage 1 choice: intergroup coop.	0.847	[0.190;1.504]	0.335	2.526	.012	*
Total intergroup cooperation (totalU)	0.043	[-0.152;0.238]	0.099	0.432	.666	
Difference in group cooperation (diffG)	0.541	[0.155;0.928]	0.197	2.745	.006	**
Round	-0.022	[-0.039;-0.005]	0.009	-2.571	.01	*
Keep × totalU	0.126	[-0.168;0.42]	0.150	0.840	.401	
Intergroup cooperation × totalU	-0.061	[-0.279;0.156]	0.111	-0.552	.581	
Keep × diffG	-0.190	[-0.767;0.387]	0.295	-0.645	.519	
Intergroup cooperation × diffG	-0.552	[-1.1;-0.003]	0.280	-1.971	.049	*
TotalU × diffG	-0.140	[-0.302;0.021]	0.082	-1.706	.088	
Keep × totalU × diffG	0.091	[-0.164;0.346]	0.130	0.702	.483	
Intergroup cooperation × totalU × diffG	0.118	[-0.077;0.313]	0.099	1.189	.234	

Note. Logistic multilevel regression; Dependent variable: 1 = Choice to meet out-group; diffG = Tokens in the other group's pool – tokens in own group pool; totalU = Total tokens in the intergroup pool; *p < .05, **p < .01, ***p < .001.

Supplementary Note 4. Identification

Figure S4 illustrates the results on the social identification. Tables S5–S7 show the results for the regression models on the difference in identification (in-group – larger collective; Table S5, see also Figure S4a), identification with the in-group (Table S6, see also Figure S4b top), and identification with the larger collective (Table S7, see also Figure S4b bottom).

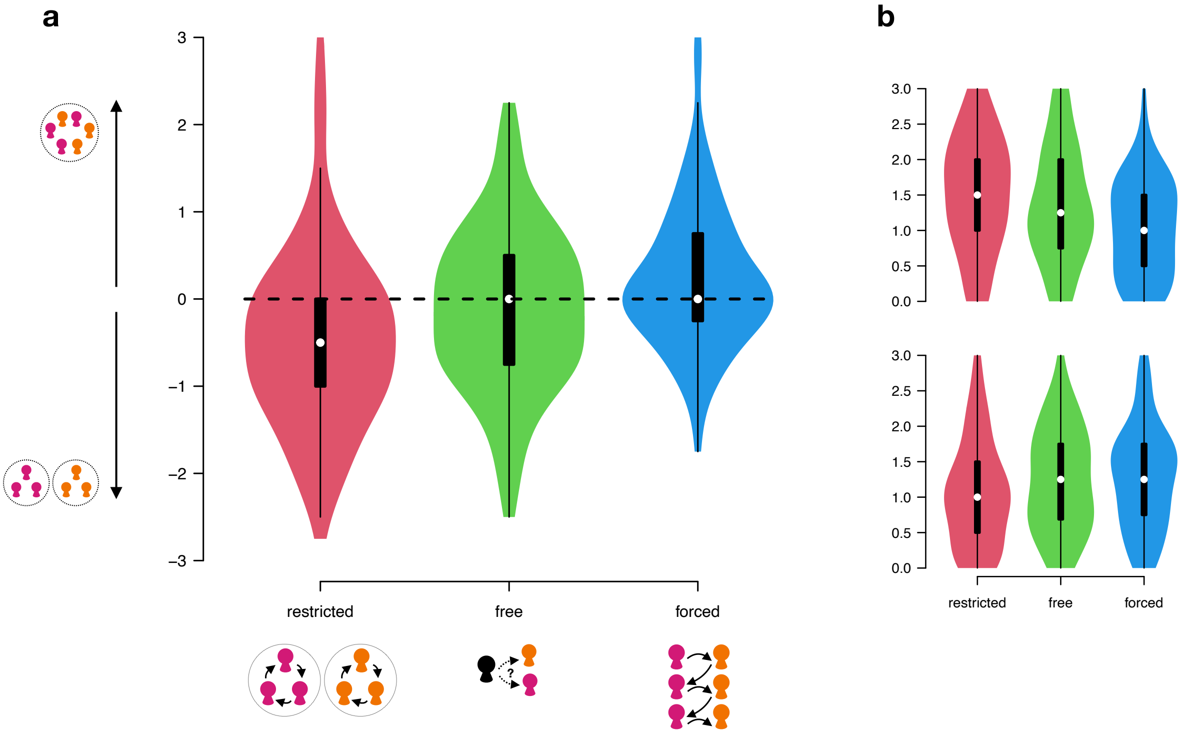

Figure S4. Social identification. After the nested mobility dilemma game, we asked each participant how much they identified with their in-group and the larger collective, allowing us to calculate a score measuring the degree to which participants identified more with the larger collective than with their in-group (i.e., collective identification – group identification). The distribution of this difference score, separated by treatment (n = 120 participants per treatment; n = 126 in the restricted mobility treatment), is shown in **a**. The white dots show the medians, and the black boxes indicate 50% of the observations. In **b,** we separately show the distribution for identification with the own group (top) and the larger collective (bottom).

Table S5. Self-reported social identification difference (in-group – larger collective) after the main task.

	b	CI	SE	z	p	
Intercept	-0.466	[-0.633;-0.3]	0.085	-5.48	<.001	***
Forced	0.687	[0.448;0.926]	0.122	5.64	<.001	***
Free	0.345	[0.107;0.584]	0.122	2.84	.006	**

Note. Linear multilevel regression; Dependent variable: Identification with everyone – identification with in-group members; *p < .05, **p < .01, ***p < .001.

Table S6. Self-reported social identification after the main task with the in-group.

	b	CI	SE	z	p	
Intercept	1.524	[1.364;1.684]	0.082	18.69	<.001	***
Forced	-0.478	[-0.707;-0.249]	0.117	-4.09	<.001	***
Free	-0.170	[-0.398;0.059]	0.117	-1.45	.152	

Note. Linear multilevel regression; dependent variable: Identification with the in-group; * <.05, ** <.01, *** <.001

Table S7. Self-reported social identification after the main task with the larger collective.

	b	CI	SE	z	p	
Intercept	1.058	[0.884;1.231]	0.089	11.94	<.001	***
Forced	0.209	[-0.039;0.458]	0.127	1.65	.105	
Free	0.176	[-0.073;0.424]	0.127	1.39	.171	

Note. Linear multilevel regression; dependent variable: Identification with the larger collective; * <.05, ** <.01, *** <.001

Supplementary Note 5. Detailed results on helping choices

Figure S5 shows a detailed breakdown of average helping rates. Helping choices may change over rounds and be driven by (i) the cooperative choices of the paired receiver, (ii) own cooperation choices, (iii) whether the receiver was a helper in the previous round or not (i.e., pertaining to second-order free-riding), and (iv) whether the receiver was part of the in- or out-group (and possibly all higher-order interactions in addition to other subject-level characteristics).

Based on Figure S5, several descriptive observations can be made. First, helping rates were quite high in general (on average, participants decided to help in 75.9% of the choices). Second, participants who decided to keep their MU in stage 1 (indicating free-riding attempts) helped the least (but still considerably; 41.8%). Third, receivers who decided to keep (i.e., free-riders) received less help. Fourth, participants who did not help in the previous round (i.e., potential second-order free-riders) also received less help than those who did. Across conditions, we therefore observe a helping pattern that (a) punishes free-riding, but also (b) punishes non-helpers. Participants therefore seemed to have used the helping stage to maintain (or enforce) cooperation in stage 1 but also maintained the enforcement device itself (i.e., stage 2) by being more likely to withhold help when paired with potential second-order free-riders.

Our main aim in the manuscript was to understand how manipulations of meeting in- or out-group members in stage 2 (under the same cost-benefit ratio of helping and information structure) influences cooperation choices in stage 1. Therefore, our experiment was not designed and does not allow to stringently reveal the different causes behind reward choices. We therefore opted to fit general regression models on the overall drivers of helping, and to fit specific regression models with the aim of testing how helping patterns shifted across our treatments (and, therefore, may explain the shift in cooperation we observed in stage 1).

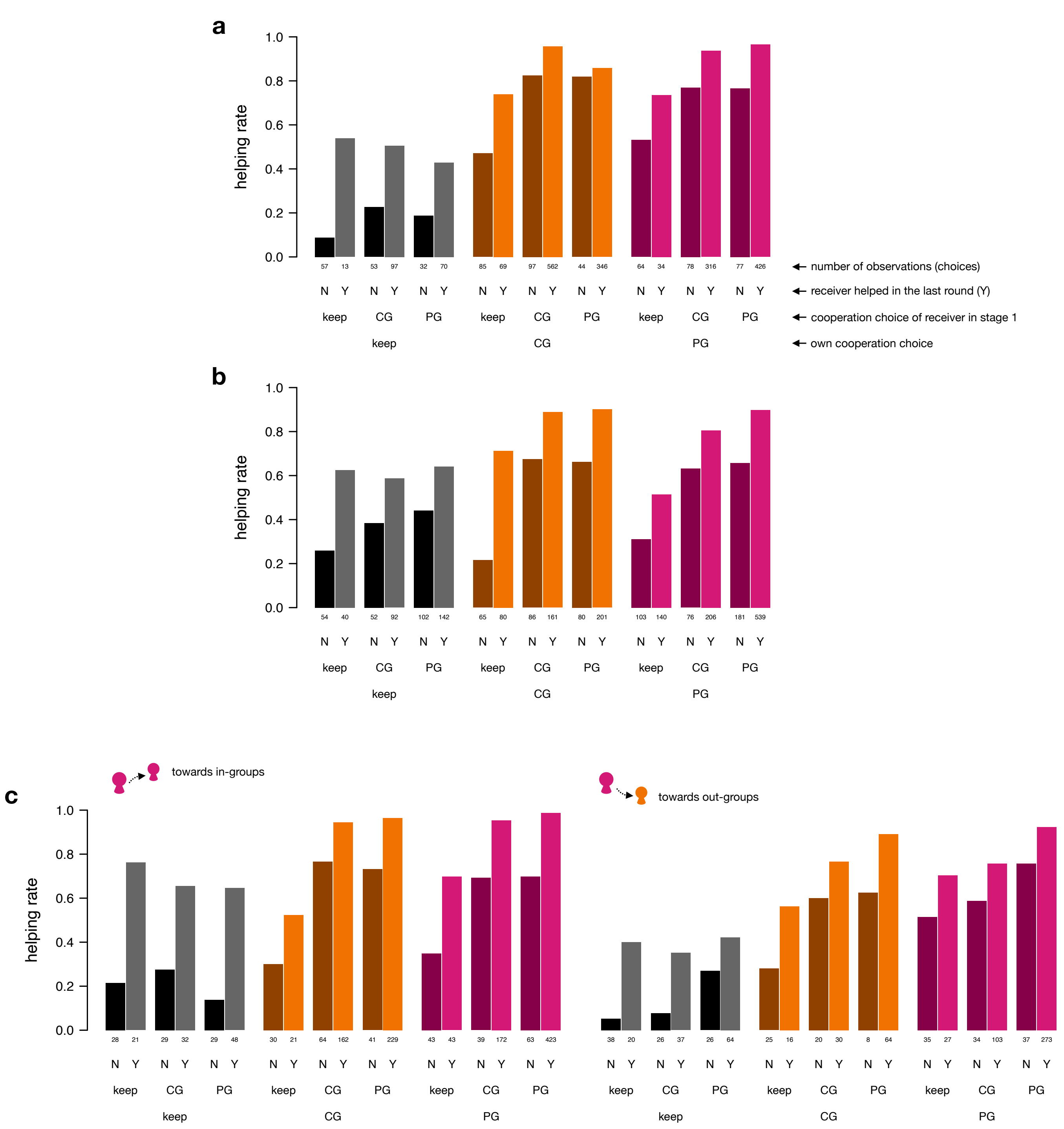

Figure S5. Helping choices. Average stage 2 helping rate, depending on whether (1) the decider kept their endowment (black/grey bars), cooperated on the club good (CG) level (orange bars), or cooperated on the public good (PG) level (pink bars); (2) the receiver kept their endowment (left two bars of each bar-group), cooperated on the CG level (middle two bars of each bar-group), or cooperated on the PG level (right two bars of each bar group); and (3) whether the receiver helped in the previous round (Y) or not (N) in the (**a**) restricted-, (**b**) forced-, or (**c**) free-mobility treatment. In the free-mobility treatment, data is further divided by whether the receiver was from the in-group (left panel) or out-group (right panel). The number below each bar indicates the number of observations (i.e., *n* observed choices).

General helping patterns. Table S8 shows the results of (logistic) regression models, predicting the decision to help in stage 2 depending on whether the receiver free-rode in stage 1 or did not help in stage 2 of the previous round (i.e., indicating possible second-order free-riding). According to these results, across all conditions, participants showed a decreased probability of helping when their receiver free-rode in stage 1 and a decreased probability of helping when their receiver did not help in the previous round, providing further evidence for the above-mentioned observations. We also explored but did not find statistical evidence for interactions between first- and second-order free-riding.

Table S8. Helping choice (stage 2) depending on whether the receiver cooperated (0) or free-rode (1) in stage 1 and whether the receiver helped in the previous stage (1) in interaction with treatments.

	***b* (model 1)**		***b* (model 2)**		***b* (model 3)**	
Intercept (partner cooperated)	3.398	***	2.120	***	2.613	***
Partner free-rode	-2.065	***			-1.860	***
Partner helped (t-1)			1.538	***	1.338	***
Free	-0.638		-0.878		-0.957	
Forced	-1.285	*	-1.242	*	-1.277	*
Round	-0.018	**	-0.042	***	-0.034	***
Partner free-rode × free	0.063				0.063	
Partner free-rode × forced	0.025				-0.141	
Partner helped (t-1) × free			0.460	*	0.500	*
Partner helped (t-1) × forced			-0.013		0.173	

Note. Logistic multilevel regression; dependent variable: 1 = helping choice; * <.05, ** <.01, *** <.001

Specific helping patterns. Next, we fitted specific regression models to test how different helping contingencies shifted across treatments. In these regressions, we control for whether

the partner helped or not in the previous stage 2 and possible interactions with treatment, since we observed these effects in the above-mentioned model.

Table S9 shows that, under free and forced mobility, participants had a higher likelihood to reward intergroup cooperation compared to the restricted-mobility treatment. Table S10 focuses only on helping choices in the free-mobility treatment and shows that helping of intergroup cooperation particularly increased when the receiver was from the out-group. Table S11 shows the results from a model that compares helping choices towards out-group members in the forced- and free-mobility treatments. Out-group members received more help when they cooperated across group boundaries, and this was significantly stronger in the free-mobility treatment.

Table S9. Helping choice (stage 2) depending on whether the receiver cooperated at the group (0) or intergroup (1) level in stage 1.

	b	CI	SE	z	p	
Intercept (partner cooperated with the group; restricted)	2.909	[2.070;3.749]	0.428	6.791	<.001	***
Partner cooperated across groups	-0.123	[-0.536;0.290]	0.211	-0.585	.559	
Free	-1.521	[-2.636;-0.407]	0.569	-2.675	.007	**
Forced	-1.740	[-2.842;-0.637]	0.562	-3.093	.002	**
Partner helped (t-1)	1.431	[0.979;1.883]	0.231	6.207	<.001	***
Round	-0.045	[-0.061;-0.029]	0.008	-5.521	<.001	***
Partner cooperated across groups × free	0.932	[0.380;1.483]	0.281	3.312	.001	**
Partner cooperated across groups × forced	0.906	[0.362;1.450]	0.278	3.263	.001	**
Free × partner helped (t-1)	0.513	[-0.072;1.099]	0.299	1.719	.086	
Forced × partner helped (t-1)	-0.005	[-0.551;0.540]	0.279	-0.020	.984	

Note. Logistic multilevel regression; dependent variable: 1 = helping choice; * <.05, ** <.01, *** <.001

Table S10. Helping choice (stage 2) depending on whether the receiver cooperated at the group (0) or intergroup (1) level in stage 1 in the free-mobility treatment.

	b	CI	SE	z	p	
Intercept (partner cooperated with the grp)	2.084	[1.231;2.936]	0.435	4.79	<.001	***
Partner cooperated across groups	0.474	[-0.025;0.972]	0.254	1.86	.063	
Out-group	-1.833	[-2.386;-1.279]	0.282	-6.49	<.001	***
Partner helped (t-1)	2.145	[1.733;2.557]	0.210	10.20	<.001	***
Round	-0.061	[-0.091;-0.031]	0.015	-4.02	<.001	***
Partner cooperated across grp × out-group	0.895	[0.182;1.607]	0.364	2.46	.014	*

Note. Logistic multilevel regression; dependent variable: 1 = helping choice; * <.05, ** <.01, *** <.001

Table S11. Helping choice (stage 2) depending on whether the receiver cooperated at the group (0) or intergroup (1) level in stage 1 when meeting out-group members in the free- or forced-mobility treatment.

	b	CI	SE	z	p	
Intercept (partner cooperated with their group; forced)	0.920	[0.294;1.546]	0.319	2.88	.004	**
Partner kept	-1.540	[-1.91;-1.171]	0.188	-8.17	<.001	***
Partner cooperated across groups	0.718	[0.387;1.049]	0.169	4.25	<.001	***
Free	-0.581	[-1.555;0.393]	0.497	-1.17	.242	
Partner helped (t-1)	1.522	[1.258;1.786]	0.135	11.29	<.001	***
Round	-0.044	[-0.062;-0.025]	0.009	-4.67	<.001	***
Partner kept × free	0.985	[0.221;1.748]	0.389	2.53	.011	*
Partner cooperated across groups × free	0.790	[0.159;1.421]	0.322	2.45	.014	*
Free × partner helped (t-1)	-0.310	[-0.879;0.26]	0.291	-1.07	.287	

Note. Logistic multilevel regression; dependent variable: 1 = helping choice; * <.05, ** <.01, *** <.001

Dynamics of intergroup rewarding. Tables S12–S14 further show a full transition matrix of choices, depending on whether the behavior was rewarded, separated by condition. Generally,

rewarding a certain form of cooperation (i.e., group or intergroup cooperation) led to an increased probability, by the rewarded person, to repeat this form of cooperation in the next round (compared to not getting rewarding; cells highlighted in bold), except for intergroup cooperation in the restricted-mobility treatment. Further, selfishness (i.e., keeping) was generally less stable, and did not systematically decrease when not being rewarded (vs. rewarded) across all treatments. It should be noted, however, that keeping was less prevalent in the first place and also less often rewarded, such that the numbers in these cells are based on fewer observations.

Table S12. Change in choices (in %) depending on previous round keep or cooperation choices (group = group cooperation, intergroup = intergroup cooperation) and whether these choices were rewarded or not (restricted-mobility treatment).

		Keep	Group	Intergroup
**Keep (t-1)**	not helped (t-1)	**34.6**	43.5	21.8
	helped (t-1)	**42.5**	44.3	13.2
**Group (t-1)**	not helped (t-1)	22.0	**54.3**	23.7
	helped (t-1)	9.0	**62.7**	28.3
**Intergroup (t-1)**	not helped (t-1)	13.6	25.0	**61.4**
	helped (t-1)	8.1	38.3	**53.6**

Table S13. Change in choices (in %) depending on previous round keep or cooperation choices (group = group cooperation, intergroup = intergroup cooperation) and whether these choices were rewarded or not (free-mobility treatment).

		Keep	Group	Intergroup
**Keep (t-1)**	not helped (t-1)	**38.3**	29.9	31.8
	helped (t-1)	**27.7**	30.9	41.4
**Group (t-1)**	not helped (t-1)	23.5	**42.9**	33.6
	helped (t-1)	11.3	**48.1**	40.6
**Intergroup (t-1)**	not helped (t-1)	21.9	23.8	**54.3**
	helped (t-1)	9.0	22.7	**68.3**

Table S14. Change in choices (in %) depending on previous round keep or cooperation choices (group = group cooperation, intergroup = intergroup cooperation) and whether these choices were rewarded or not (forced-mobility treatment).

		Keep	Group	Intergroup
**Keep (t-1)**	not helped (t-1)	**36.3**	30.7	33.1
	helped (t-1)	**37.9**	23.8	38.3
**Group (t-1)**	not helped (t-1)	31.6	**32.0**	36.4
	helped (t-1)	12.8	**43.9**	43.3
**Intergroup (t-1)**	not helped (t-1)	22.8	25.3	**51.9**
	helped (t-1)	10.0	27.4	**62.6**

Helping dynamics in the free-mobility treatment. Figure S6 shows the patterns of rewarding group vs. intergroup cooperation when paired with in-group (S6a) or out-group members (S6b) in the free-mobility treatment. While for in-group members, the average propensity to receive help was mostly independent of whether the target cooperated at the group or intergroup level and did not significantly change over rounds, participants who voluntarily decided to be paired with out-group members more strongly enforced intergroup compared to group cooperation.

Descriptively, we also observed a slight downward trend in helping group cooperators when they belonged to the out-group. Table S15 shows the regression results. Similar to the results from Table S10, we observed lower rates of helping group cooperators when the target belonged to the out-group ('out-group' coefficient) and increased helping of intergroup cooperators (intergroup cooperation × out-group). We did not observe any significant effects over the rounds or interactions with rounds. Hence, there was no statistical support that these helping patterns changed throughout the course of the experiment.

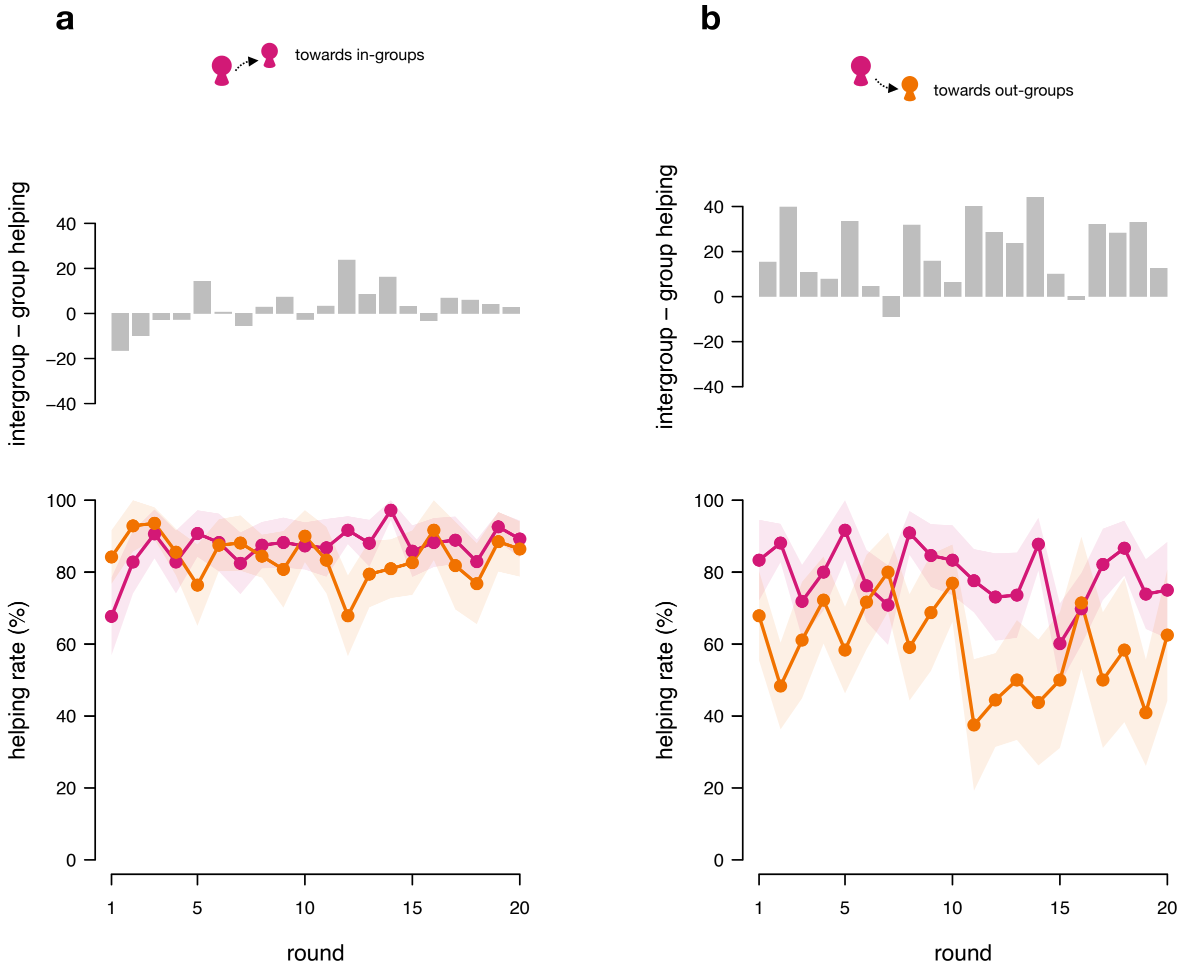

Figure S6. Helping dynamics across rounds in the free-mobility treatment (n = 20 groups). Bottom: Average helping of group cooperation (orange line) and intergroup cooperation (pink line) across rounds when meeting in-group members (**a**) or out-group members (**b**). Top: Difference in helping intergroup and in-group cooperation across rounds when meeting in-group members (**a**) or meeting out-group members (**b**). Positive values indicate a higher propensity to help intergroup rather than group cooperation. Error bands indicate the standard errors of the round means.

Table S15. Helping choices (stage 2) depending on stage 1 cooperation decisions, group membership and round in the free-mobility treatment.

	b	CI	SE	z	p	
Intercept (group cooperation; in-group)	3.123	[2.161;4.085]	0.491	6.364	<.001	***
Intergroup cooperation	0.151	[-0.664;0.966]	0.416	0.364	.716	
Out-group	-1.573	[-2.404;-0.741]	0.424	-3.705	<.001	***
Round	-0.039	[-0.091;0.012]	0.026	-1.493	.136	
Intergroup cooperation × out-group	1.316	[0.148;2.484]	0.596	2.208	.027	*
Intergroup cooperation × round	0.039	[-0.033;0.11]	0.036	1.061	.289	
Out-group × round	0.000	[-0.079;0.08]	0.041	0.005	.996	
Intergrp cooperation × out-group × round	-0.033	[-0.139;0.073]	0.054	-0.605	.545	

Note. Logistic multilevel regression; dependent variable: 1 = helping choice; * <.05, ** <.01, *** <.001

Supplementary Note 6. Additional results on social preferences

Figure S7 shows the bivariate associations of social preferences (as measured with the social value orientation slider (SVO) measure; see Methods) with cooperation, and helping choices. Based on the results of linear multilevel regression models, social preferences were positively related to the proportion of intergroup cooperation choices ($p < .001$) and the decision to reward/help in stage 2 ($p < .001$), but negatively to keeping tokens ($p < .001$), irrespective of the treatment. Social preferences were not significantly related to group cooperation ($p = .942$) across all treatments, in line with [38,82]. Social preferences also did not predict the choice to meet in-group vs. out-group members ($p = .630$) in the free-mobility treatment.

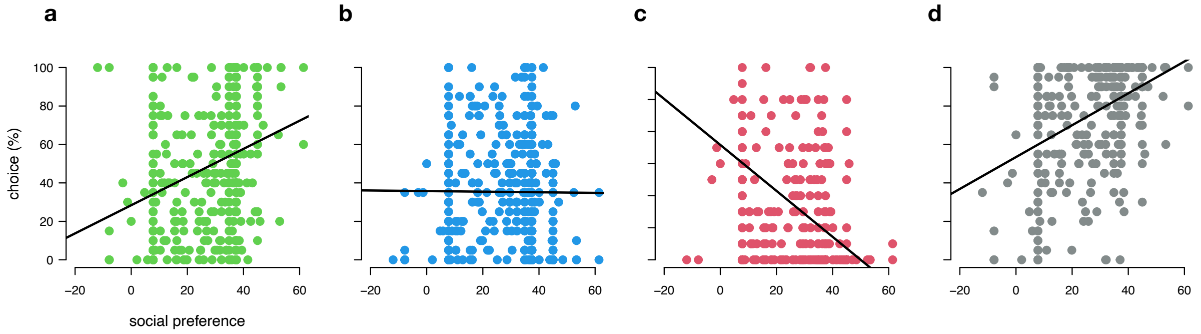

Figure S7. Social preferences. Association of social preferences (social value orientation angle) and the average choice to (a) cooperate across groups, (b) parochially, or (c) free-ride, and (d) the decision to help in stage 2. Dots are individual participants (n = 366 participants per panel); lines show the best linear fit.

Table S16 shows how a higher SVO angle is related to more intergroup cooperation, irrespective of the treatment. Hence, according to the model, participants with higher social preferences tended to cooperate more at the intergroup level, potentially explaining why intergroup cooperation, while decreasing over rounds (see Figure 2b and Table S2), is not zero in the restricted-mobility treatment.

Table S16. Intergroup cooperation as a function of individual-level social preferences (SVO angle) and treatment.

	b	CI	SE	z	p	
Intercept (restricted-mobility treatment)	0.172	[0.039;0.305]	0.068	2.537	.012	*
SVO angle	0.008	[0.004;0.012]	0.002	4.215	<.001	***
Free-mobility treatment	0.192	[0.008;0.376]	0.094	2.042	.042	*
Forced-mobility treatment	0.203	[0.017;0.39]	0.095	2.136	.034	*
SVO angle × free mobility	-0.001	[-0.006;0.004]	0.003	-0.515	.607	
SVO angle × forced mobility	-0.002	[-0.008;0.003]	0.003	-0.922	.357	

Note. Linear multilevel regression; dependent variable: 1 = universal cooperation; * <.05, ** <.01, *** <.001

Supplementary Note 7. Additional results on motives

After the nested mobility dilemma game, we asked participants in the free-mobility treatment to report their reasons for choosing in-group or out-group members in stage 2. Tables S17 and S18 summarize the proportion of answers that participants gave for choosing out-group and in-group members, respectively. As a reason for meeting out-group members, most participants reported that they "[…] wanted to motivate the members of the other group to invest in the intergroup pool" (33%). As a reason for meeting in-group members, most participants reported that their "[…] group was more cooperative than the other group" (32%).

Table S17. Proportion of self-reported motives behind the choice to meet out-group members in the free-mobility treatment.

**Motive**	**Wording**	**Proportion**
**Reduce borders**	"I wanted to break through group boundaries"	**24%**
**Membership**	"I wanted to show that belonging to a group is less important to me"	**9%**
**Cooperation difference**	"I had the impression that the other group was more cooperative than mine"	**31%**
**Enforcement of intergroup cooperation**	"I wanted to motivate the members of the other group to invest in the intergroup pool"	**33%**
**Enforcement of group cooperation**	"I wanted to motivate the members of the other group to invest in their group pool"	**3%**

Table S18. Proportion of self-reported motives behind the choice to meet in-group members in the free-mobility treatment.

**Motive**	**Wording**	**Proportion**
**Enforce borders**	"I wanted to help maximize the profit within my group"	**22%**
**Membership**	"I wanted to show that belonging to a group is important to me"	**14%**
**Cooperation difference**	"I had the impression that my group was more cooperative than the other group"	**32%**
**Enforcement of intergroup cooperation**	"I wanted to motivate the members of my group to invest in the intergroup pool"	**18%**
**Enforcement of group cooperation**	"I wanted to motivate the members of my group to invest in our group pool"	**14%**
