# Supplementary material for: Free mobility across group boundaries promotes intergroup cooperation
Source: Commun Psychol. 2025 Jan 25;3:10. doi: 10.1038/s44271-025-00192-y (PMC11762412; doi:10.1038/s44271-025-00192-y)
Supplement: Supplementary file 3 — Description of Additional Supplementary Files [file 44271_2025_192_MOESM3_ESM.pdf]

Additional Supplementary Files

Supplementary Data 1. Processed dataset of the study. Each line indicates one recorded decision per participant in the respective round. Treatment is coded as “own” = “restricted mobility”; “other” = “forced mobility”; “endo” = “free mobility”.
