# Supplementary material for: Free mobility across group boundaries promotes intergroup cooperation
Source: Commun Psychol. 2025 Jan 25;3:10. doi: 10.1038/s44271-025-00192-y (PMC11762412; doi:10.1038/s44271-025-00192-y)
Supplement: Supplementary file 5 — Reporting Summary [file 44271_2025_192_MOESM5_ESM.pdf]

nature portfolio

Corresponding author(s): Joerg Gross

Last updated by author(s): Aug 15, 2024

Reporting Summary

Nature Portfolio wishes to improve the reproducibility of the work that we publish. This form provides structure for consistency and transparency in reporting. For further information on Nature Portfolio policies, see our Editorial Policies and the Editorial Policy Checklist.

Statistics

For all statistical analyses, confirm that the following items are present in the figure legend, table legend, main text, or Methods section.

n/a	Confirmed	
☐	☒	The exact sample size (*n*) for each experimental group/condition, given as a discrete number and unit of measurement
☐	☒	A statement on whether measurements were taken from distinct samples or whether the same sample was measured repeatedly
☐	☒	The statistical test(s) used AND whether they are one- or two-sided<br>*Only common tests should be described solely by name; describe more complex techniques in the Methods section.*
☐	☒	A description of all covariates tested
☐	☒	A description of any assumptions or corrections, such as tests of normality and adjustment for multiple comparisons
☐	☒	A full description of the statistical parameters including central tendency (e.g. means) or other basic estimates (e.g. regression coefficient) AND variation (e.g. standard deviation) or associated estimates of uncertainty (e.g. confidence intervals)
☐	☒	For null hypothesis testing, the test statistic (e.g. *F*, *t*, *r*) with confidence intervals, effect sizes, degrees of freedom and *P* value noted<br>*Give P values as exact values whenever suitable.*
☒	☐	For Bayesian analysis, information on the choice of priors and Markov chain Monte Carlo settings
☐	☒	For hierarchical and complex designs, identification of the appropriate level for tests and full reporting of outcomes
☐	☒	Estimates of effect sizes (e.g. Cohen's *d*, Pearson's *r*), indicating how they were calculated

Our web collection on statistics for biologists contains articles on many of the points above.

Software and code

Policy information about availability of computer code

Data collection	custom code written in PHP/JavaScript
Data analysis	Performed in R version 4.2.2

For manuscripts utilizing custom algorithms or software that are central to the research but not yet described in published literature, software must be made available to editors and reviewers. We strongly encourage code deposition in a community repository (e.g. GitHub). See the Nature Portfolio guidelines for submitting code & software for further information.

Data

Policy information about availability of data

All manuscripts must include a data availability statement. This statement should provide the following information, where applicable:

- Accession codes, unique identifiers, or web links for publicly available datasets
- A description of any restrictions on data availability
- For clinical datasets or third party data, please ensure that the statement adheres to our policy

Data, materials, and analyses can be accessed at: https://osf.io/j634g/?view_only=606440f2eb334c0a90e19da4f22d17b8

Human research participants

Policy information about studies involving human research participants and Sex and Gender in Research.

Reporting on sex and gender	Participants self-reported their gender at the end of the study. We report the proportion of self-identified female participants (55%) in the sample. Gender was neither pre-registered nor considered as a variable of interest in the study and, hence, not further analysed.
Population characteristics	Other than age > 15, we had no inclusion or exclusion criteria regarding population characteristics.
Recruitment	Participants were recruited from the subject pool of the University of Zurich (mostly consisting of university students).
Ethics oversight	Department of Psychology, University of Zurich

Note that full information on the approval of the study protocol must also be provided in the manuscript.

Field-specific reporting

Please select the one below that is the best fit for your research. If you are not sure, read the appropriate sections before making your selection.

☐ Life sciences ☒ Behavioural & social sciences ☐ Ecological, evolutionary & environmental sciences

For a reference copy of the document with all sections, see nature.com/documents/nr-reporting-summary-flat.pdf

Behavioural & social sciences study design

All studies must disclose on these points even when the disclosure is negative.

Study description	Laboratory experiment with three independent conditions to which participants were assigned randomly (collecting quantitative data; i.e., decisions taken by participants).
Research sample	Undergraduate students from the University of Zurich. Sample is not representative. Experimental manipulations are aimed to derive causal effects within this sample.
Sampling strategy	Participants, registered in the recruitment system of the University Zurich, were randomly invited to take part in the study. Sample size was pre-registered and based on previous studies with similar design features.
Data collection	Each participant was assigned their own computer in a separate cubicle. Participants read instructions and made decisions on their computer. Participants within each session were randomly assigned to treatments such that experimenters were not fully aware of the treatment they were assigned to.
Timing	Start: 2023.04.21; End: 2023.10.27
Data exclusions	No data were excluded from the analyses
Non-participation	No participants declined to participate or dropped out. Since we needed fixed-size groups for the study, it happened that some people that signed up for the study could not take part in the study and were sent home with a showup fee (and the possibility to sign up for a future session).
Randomization	Participants were randomly allocated to groups and treatments.

Reporting for specific materials, systems and methods

We require information from authors about some types of materials, experimental systems and methods used in many studies. Here, indicate whether each material, system or method listed is relevant to your study. If you are not sure if a list item applies to your research, read the appropriate section before selecting a response.

Materials & experimental systems

n/a	Involved in the study
☒	☐ Antibodies
☒	☐ Eukaryotic cell lines
☒	☐ Palaeontology and archaeology
☒	☐ Animals and other organisms
☒	☐ Clinical data
☒	☐ Dual use research of concern

Methods

n/a	Involved in the study
☒	☐ ChIP-seq
☒	☐ Flow cytometry
☒	☐ MRI-based neuroimaging
